# Intrinsic patient factors predictive of inpatient rehabilitation facility discharge following primary total knee arthroplasty: a systematic review and meta-analysis

**DOI:** 10.1186/s12891-020-03499-5

**Published:** 2020-07-22

**Authors:** Larissa Sattler, Wayne Hing, Evelyne Rathbone, Christopher Vertullo

**Affiliations:** 1 Bond University, Bond Institute of Health and Sport, Promethean Way, Robina, QLD 4226 Australia; 2 Knee Research Australia, 8-10 Carrara Street, Benowa, QLD 4217 Australia

**Keywords:** Total knee arthroplasty (TKA), Rehabilitation, Discharge, Predictors, Systematic review, Meta-analysis

## Abstract

**Background:**

Total Knee Arthroplasty (TKA) reduces pain and improves function in those suffering from severe osteoarthritis. A significant cost of TKA is post-acute care, however, current evidence suggests that discharge to an Inpatient Rehabilitation Facility (IRF) has inferior outcomes to home discharge, with no greater benefit in physical function. Only individual studies have investigated TKA patient characteristics predictive of discharge destination, therefore, the aim is to systematically review the literature and meta-analyse intrinsic patient factors predictive of IRF discharge. If predictive factors are known, then early discharge planning and intervention strategies could be implemented.

**Methods:**

Databases PubMed, CINAHL, Embase, Cochrane, and Pedro were searched up to October 2019 for all studies investigating pre-operative intrinsic patient factors predictive of IRF discharge. For assessing the methodological quality of included studies, the Quality In Prognosis Studies (QUIPS) tool was used. Statistical analysis and graphical reporting were conducted in R statistical software. To assess the effect of predictors of discharge destination, odds ratios with the corresponding 95%CI were extracted from the results of univariate and multivariable analyses.

**Results:**

A total of 9 articles published between 2011 to 2018 with 218,151 TKA patients were included. Of the 13 intrinsic patient factors reported, 6 met the criteria for synthesised review: age, obesity, comorbidity, gender, SF-12/VR-12 survey, and smoking. Due to the heterogeneity of statistical analysis and reporting 2 variables could undergo meta-analysis, gender and smoking. Female gender increased the likelihood of IRF discharge by 78% (OR = 1.78; 95%CI = 1.43–2.20; I2 = 33.3%), however, the relationship between smoking status and discharge destination was less certain (OR = 0.80; 95%CI = 0.42–1.50; I2 = 68.5%).

**Conclusion:**

In this systematic literature review and meta-analysis female gender was shown to be predictive of IRF discharge after total knee arthroplasty. There was also a trend for those of older age and increased comorbidity, as measured by the Charlson Comorbidity Index, or the severely obese to have an increased likelihood of IRF discharge. The marked heterogeneity of statistical methods and reporting in existing literature made pooled analysis challenging for intrinsic patient factors predictive of IRF discharge after TKA. Further, high quality studies of prospective design on predictive factors are warranted, to enable early discharge planning and optimise resource allocation on post-acute care following TKA.

**Trial registration:**

This review was registered with PROSPERO (CRD42019134422).

## Background

From 2014 to 2030 in the United States, primary total knee arthroplasty (TKA) is projected to increase by 85% to 1.26 million surgical procedures [1]. TKA is widely regarded as a cost-effective intervention for end-stage knee osteoarthritis, improving both a patient’s functional status and overall health quality [2]. However, with the societal burden of cost for TKA increasing, there is a need to evaluate the economic efficiency of current models of care [3, 4].

Total Joint Arthroplasty (TJA) is reportedly the most frequent procedure leading to post-acute admission to an Inpatient Rehabilitation Facility (IRF), representing one of the most significant costs associated with TKA [4, 5]. Acute post-discharge care following primary TKA can account for up to 37% of the total procedure cost, with home discharge reported as costing $16,000 less than discharge to an IRF in the United States [6, 7]. While IRF patients receive multidisciplinary input such as physical therapy and occupational therapy, this has yet to translate into evidence for improved functional outcomes when compared with TKA patients discharged directly to home [8, 9]. Moreover, retrospective studies relying on administrative datasets have shown TKA patients with an IRF discharge have a significantly higher adverse event and 30-day readmission rate compared to those discharged home [10, 11].

The decision to discharge a patient to an IRF is dependent on many variables and identifying pre-operative factors that increase the likelihood of IRF discharge will better facilitate pre-operative discharge planning and resource allocation. Given an estimated 1.82 billion is spent on IRF discharge after lower-extremity arthroplasty it is imperative to determine which factors create a higher risk for non-home discharge [12]. To date, only individual studies have investigated TKA patient characteristics predictive of discharge destination, therefore, the aim of this review was to systematically review the literature and conduct a meta-analysis on reported intrinsic patient factors predictive of IRF discharge.

## Methods

This systematic review and meta-analysis was prospectively registered on PROSPERO (International prospective register of systematic reviews), registration CRD42019134422 and is reported in accordance with the guidelines from the PRISMA (Preferred Reporting Items for Systematic Reviews and Meta-Analyses) statement [13].

### Search strategy

Relevant online databases PubMed, Cumulative Index to Nursing and Allied Health (CINAHL), Embase, Cochrane, and Pedro were systematically searched from database inception to October 3rd, 2019. Key terms were identified for the search, including knee arthroplasty, predictor, and discharge, as well as synonym words, utilising Medical Subject Heading and Boolean operator terms. The complete search strategy is reported in Table 1.

**Table 1:** Critical review databases and search terms

Database	Search Terms				
PubMed CINAHL Embase COCHRANE PEDro	“Arthroplasty, Replacement, Knee” (MESH) OR Knee Replacement OR TKR	AND	Predict* OR Determin* OR Preoperative OR Factors OR Characteristic* OR Influence OR Affects	AND	Discharge* OR “Patient Discharge”[Mesh])

* = truncation search

### Study eligibility

We included all articles of any study design investigating pre-operative patient factors for their level of predictivity of IRF discharge following primary TKA. Key inclusion criteria were that the article be available in full text English, a search term was required in the title or abstract, the population studied was primary unilateral knee replacement patients, a pre-operative intrinsic patient factor was a variable in the study and an outcome included discharge destination. Intrinsic factors were defined as those inherent to the individual, including demographic characteristics age, gender, ethnicity, socioeconomic status as well as clinical factors such as presence of co-morbidity. Behavioural factors such as smoking and alcohol consumption, and patient reported outcome measures that capture the patient’s perspective were also considered intrinsic factors and included. Non-intrinsic factors were excluded, where the patient was subject to an intervention such as participation in a pre-operative exercise class or education session. Additional exclusion criteria were post-operative variables including length of stay (LOS), readmission status and other post-operative complications. Studies that did not separate the reporting of primary TKA were also excluded, as were studies that took place after discharge from the post-acute hospital setting, such as outpatient or inpatient rehabilitation.

### Study selection

Based on the inclusion criteria, an initial screening of titles and abstracts was conducted, next, a screening of extracted full text papers was conducted for final review. Those studies that met the inclusion/exclusion criteria for this literature review were then screened for eligibility to be included for meta-analysis.

### Data extraction

A modified form based on the Critical Appraisal and Data Extraction for Systematic Reviews of Prediction Modelling Studies (CHARMS) Checklist was used for data extraction [14]. Data regarding author, year of publication, country, study design, exclusion criteria, patient factors investigated, sample size, participant age and gender as well as a description of statistical analysis undertaken were included.

### Quality assessment

For assessing the quality of individual studies, the Quality In Prognosis Studies (QUIPS) tool was applied [15]. The QUIPS tool is a validated tool for assessing risk of bias in prognostic factor studies and provides a qualitative assessment of six domains: (I) Study Participants, (II) Study Attrition, (III) Prognostic Factor Measurement, (IV) Outcome Measurement, (V) Study Confounding and (VI) Statistical Analysis and Reporting [16]. For each of these 6 domains, the responses `yes’, `partial’, `no’ or `unsure’ for three up to seven items within each domain are combined to assess the risk of bias. Two reviewers, following the guidelines of Hayden et al. 2013 [16] independently assessed each study, ranking the risk of bias as high, moderate or low. If the authors disagreed on the risk of bias rating, a consensus agreement was reached by joint discussion.

### Statistical analysis

Statistical analysis and graphical reporting were conducted in R statistical software, version 3.5.3 [R-Core], using packages metafor and forestplot [17]. To assess the effect of predictors of discharge destination, odds ratios with the corresponding 95%CI were extracted from the results of univariate and multivariable analyses. When these effect sizes were not reported in univariate results, they were computed from the count data, if available.

Meta-analysis of a patient factor was considered where there was a minimum of three studies reporting an association between the predictor and discharge destination. Meta-analysis was only applied to a factor if the reference categories were similar. Forest plots without pooled effect were produced for those studies considered ineligible for meta-analysis to gain insight into the degree of predictability of the patient factor. Heterogeneity of included studies’ estimates were assessed by computing the *I*^*2*^ statistic and was considered statistically significant at *P* < 0.10. *I*^*2*^ values were used to describe the percentage of total variation across studies; an *I*^*2*^ value of 25% was considered low, 50% moderate, and 75% high [18]. Pooling of the odds ratios across studies was carried out with a random-effects model using the inverse-variance method.

## Results

### Literature search and study characteristics

**Fig. 1:**
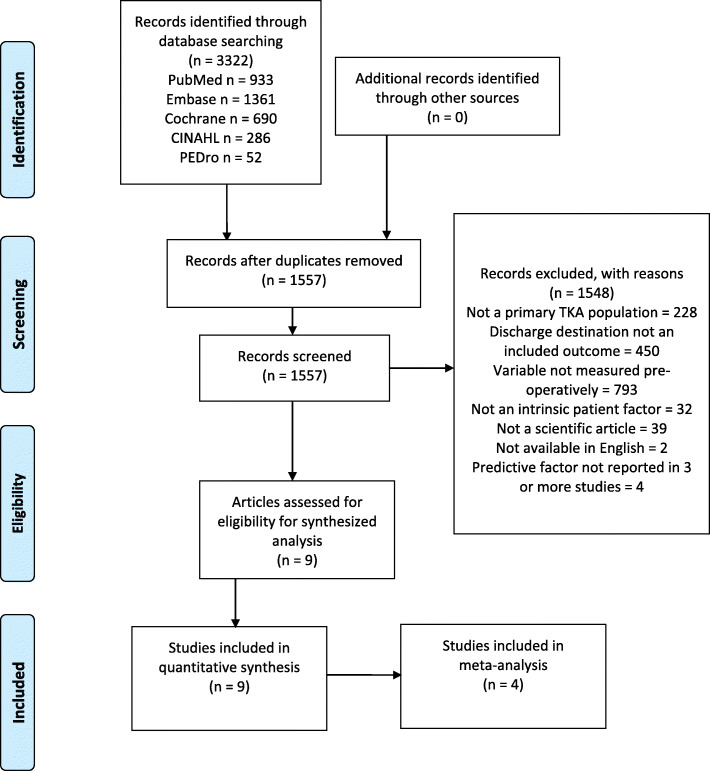
Prisma Flow Diagram of systematic search, screening and selection process

The results of the search strategy and screening process are shown as a flowchart in Fig. 1. After duplicates were removed, 1557 articles were screened for eligibility with reasons for exclusion listed. A total of 9 articles published between 2011 to 2018, with 218,151 TKA patients, were included in this review [19–27]. Of those, 4 articles [22–24, 27] met the criteria to undergo meta-analysis.

The individual studies and their characteristics can be found in Table 2. Patient demographics were similar across studies for mean age ranging from 61 to 70 years, however, female gender had greater variation, ranging from 56 to 83% of the study populations. Country of origin was the United States for 8 of the 9 papers, with 1 being from Australia. All studies were of observational cohort design, 7 were performed retrospectively.

**Table 2:** Study Characteristics

Author, Year. Country	Study Title	Study design; Exclusion criteria (if provided)	Patient Factors^**a**^	TKA Patients (***N***)	Age: mean (±SD or range)	Female Gender: ***n*** (%)	Statistical Analysis^**a**^
Anoushiravani et al., 2016. USA	Assessing In-Hospital Outcomes and Resource Utilization After Primary Total Joint Arthroplasty Among Underweight Patients.	Retrospective matched cohort.; Weight loss and obesity, due to the nature of the study, were excluded from the matching criteria.	BMI	1315	70 (15–91	1029 (78)	Univariate
Crawford et al., 2011. USA	Preoperative Predictors of Length of Hospital Stay and Discharge Disposition Following Primary Total Knee Arthroplasty at a Military Medical Center.	Retrospective cohort; Bilateral, revision, or uni-compartmental TKA.	Age ASA BMI	383	64 (±10)	214 (56)	Univariate and Multivariable regression
D’Apuzzo et al., 2015. USA	The John Insall Award: Morbid Obesity Independently Impacts Complications, Mortality, and Resource Use After TKA.	Retrospective matched cohort; Obesity, due to the nature of the study, was excluded from the matching criteria. Morbidly obese patients who could not be matched were excluded.	BMI	180,585	61 (22–90)	135,541 (75)	Univariate
Murphy et al., 2018. Australia	The Impact of Older Age on Patient Outcomes Following Primary Total Knee Arthroplasty.	Retrospective cohort	Age ASA BMI CCI Gender SF-12 PROM SES Smoking	2838	70 (± 9)	1882 (66)	Univariate and Multivariable regression
Prohaska et al., 2017. USA	Preoperative Body Mass Index and Physical Function are Associated with Length of Stay and Facility Discharge after Total Knee Arthroplasty	Prospective cohort; Bilateral procedures, simultaneous and staged within one year, and those with concomitant joint arthroplasty or ligament repair on the ipsilateral extremity were excluded.	Age BMI CCI Gender Hemoglobin Smoking VR-12 PROM	716	63 (±11)	425 (59)	Univariate and Multivariable regression
Rissman et al., 2016. USA	Predictors of Facility Discharge, Range of Motion, and Patient-Reported Physical Function Improvement After Primary Total Knee Arthroplasty: A Prospective Cohort Analysis	Prospective cohort; Simultaneous bilateral TKAs were excluded.	Age BMI CCI Gender ROM VR-12 PROM	738	64 (±10)	422 (57)	Univariate and Multivariable regression
Sayeed et al. 2016. USA	Comparing In-Hospital Total Joint Arthroplasty Outcomes and Resource Consumption Among Underweight and Morbidly Obese Patients	Retrospective matched cohort; Weight loss and obesity, due to the nature of the study, were excluded from the matching criteria.	BMI	956	67 (15–91)	791 (83)	Univariate
Schwarzkopf et al., 2016. USA	Factors Influencing Discharge Destination After Total Knee Arthroplasty: A Database Analysis	Retrospective cohort	Age CCI Gender Ethnicity	28,611	68	17,930 (63)	Multinomial regression
Sikora-Klak et al., 2016. USA	The Effect of Comorbidities on Discharge Disposition and Readmission for Total Joint Arthroplasty Patients	Retrospective cohort Bilateral procedures were excluded as were patients undergoing joint arthroplasty for fracture.	Age BMI Diabetes Gender Smoking VTE history	2009	65 (±11)	1347 (67)	Univariate and Multivariable regression

*Abbreviations*: *ASA* American society of anesthesiologists, *BMI* body mass index (kg/m2), *CCI* Charlson comorbidity index, *Hb* Hemoglobin, *ROM* range of motion, *SES* socioeconomic status, *SF-12* 12 item Short Form Health Survey (physical component score), *VR-12* Veterans RAND 12 Item Health Survey, *VTE* Venous thromboembolism

^a^*Predictors* and *Statistical Analysis* are in reference to the outcome of interest, Discharge Destination

### Methodical quality

QUIPS ranking did not vary by more than one category between raters for any criteria for each publication and consensus was achieved by discussion. Table 3 presents the risk of bias scores for all included studies. The risk of bias was ranked low across all studies for “study participation,” “prognostic factor measurement,” and “outcome measurement.” However, “study confounding,” was ranked moderate or high for risk of bias across 6 of the 9 studies. As 8 of the 9 studies did not report on perioperative factors such as surgical and anaesthetic technique or physical therapy protocols this could have a confounding effect on the other patient factors assessed.

**Table 3:** Results of risk of bias assessment using the Quality in Prognosis Studies (QUIPS) tool for included studies

Study	Study participation	Study attrition	Prognostic factor measurement	Outcome measurement	Study confounding	Statistical analysis and reporting
Anoushiravani	Low	Low	Low	Low	Moderate	Low
Crawford	Low	Low	Low	Low	Moderate	Moderate
D’Apuzzo	Low	Low	Low	Low	Moderate	Low
Murphy	Low	Low	Low	Low	Low	Low
Prohaska	Low	Low	Low	Low	Low	Low
Rissman	Low	Low	Low	Low	Low	Low
Sayeed	Low	Low	Low	Low	Moderate	Moderate
Schwarzkopf	Low	Moderate	Low	Low	High	Low
Sikora-Klak	Low	Low	Low	Low	Moderate	Low

Study participation = the representativeness of the study sample; Study attrition = whether participants with follow-up data represent persons enrolled in the study; Prognostic factor measurement = adequacy of prognostic factor measurement; Outcome measurement = adequacy of outcome measurement; Study confounding = potential confounding factors; Statistical analysis and reporting = the appropriateness of the statistical analysis and completeness of reporting

### Intrinsic patient factors analysed

Patient factors in the included studies that were analysed for their association with discharge destination were age, American society of anaesthesiology (ASA) score, body mass index (BMI), Charlson comorbidity index (CCI), diabetes, gender, ethnicity, haemoglobin (Hb), knee range of motion (ROM), socioeconomic status (SES), 12 item short form health survey or 12 item Veteran’s RAND health survey (SF-12/VR-12), smoking and venous thromboembolism (VTE) history. Table 4 details the predictability of each patient factor on discharge destination for the included studies. Of the 13 patient factors reported on, 6 factors met the criteria for comparison and a synthesised review, these were age, BMI, CCI, gender, SF-12/VR-12 survey, and smoking status.

**Table 4:** Intrinsic Patient Factors Predictive of Inpatient Rehabilitation Discharge

	Anoushiravani	Crawford	D’Apuzzo	Murphy	Prohaska	Rissman	Sayeed	Schwarzkopf	Sikora-Klak	Total
**Age (older)**	–	✓✓	–	✓✓	✓✓	✓✓	–	✓✓ ^b^	✓✓	6/6
**ASA (higher)**	–	✓✓	–	✓	–	–	–	–	–	2/2
**BMI (higher)**	✓ ^a,b^	x^b^	✓^b^	✓✓	✓✓	✓✓	x^b^	–	–	5/7
**CCI (higher)**	–	–	–	✓✓	✓✓	✓✓	–	✓✓^b^		4/4
**Diabetes (yes)**	–	–	–	–	–	–	–	–	✓✓	1/1
**Gender (Female)**	–	–	–	✓✓	✓✓	✓✓	–	✓✓^b^	✓✓	5/5
**Ethnicity (non-caucasian)**	–	–	–	–	–	–	–	✓✓	–	1/1
**Hb (lower)**	–	–	–	–	✓✓	–	–	–	–	1/1
**Knee ROM (lower)**	–	–	–	–	–	x	–	–	–	0/1
**SES (lower)**	–	–	–	✓✓	–	–	–	–	–	1/1
**SF-12/VR-12 (lower)**	–	–	–	✓✓	✓✓	✓✓	–	–	–	3/3
**Smoking (yes)**	–	–	–	✓✓	x	–	–	–	✓	2/3
**VTE History (yes)**	–	–	–	–	–	–	–	–	✓✓	1/1

*Abbreviations*: *ASA* American society of anesthesiologists, *BMI* body mass index (kg/m^2^), *CCI* Charlson comorbidity index, *Hb* Hemoglobin, *ROM* range of motion, *SES* socioeconomic status, *SF-12* 12 item Short Form Health Survey (physical component score), *VR-12* Veterans RAND 12 Item Health Survey, *VTE* Venous thromboembolism

^a^BMI < 19 kg/m^2^ (Underweight patients)

^b^Factor not able to undergo pooled analysis due to statistical reporting heterogeneity

✓✓ = Factor significant in multivariable analysis ✓ = Factor only significant in univariate analysis x = Factor not significant in univariate analysis – indicates that a factor was not assessed

#### Demographic factors

**Fig. 2:**
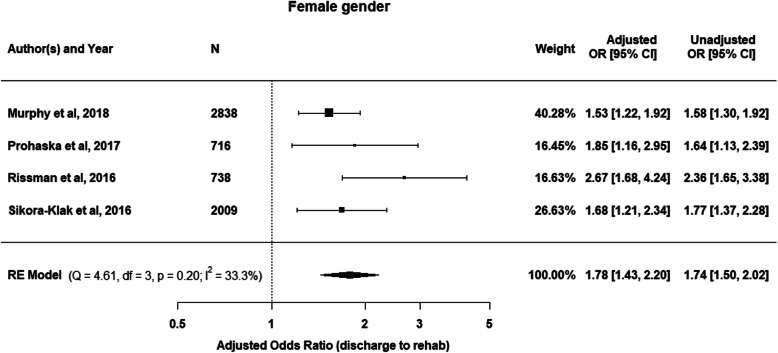
Meta-analysis showing the adjusted odds ratios and 95%CI of a random effects (RE) model for likelihood of discharge to IRF for females compared to males. Unadjusted odds ratios with 95%CI are also reported

**Fig. 3:**
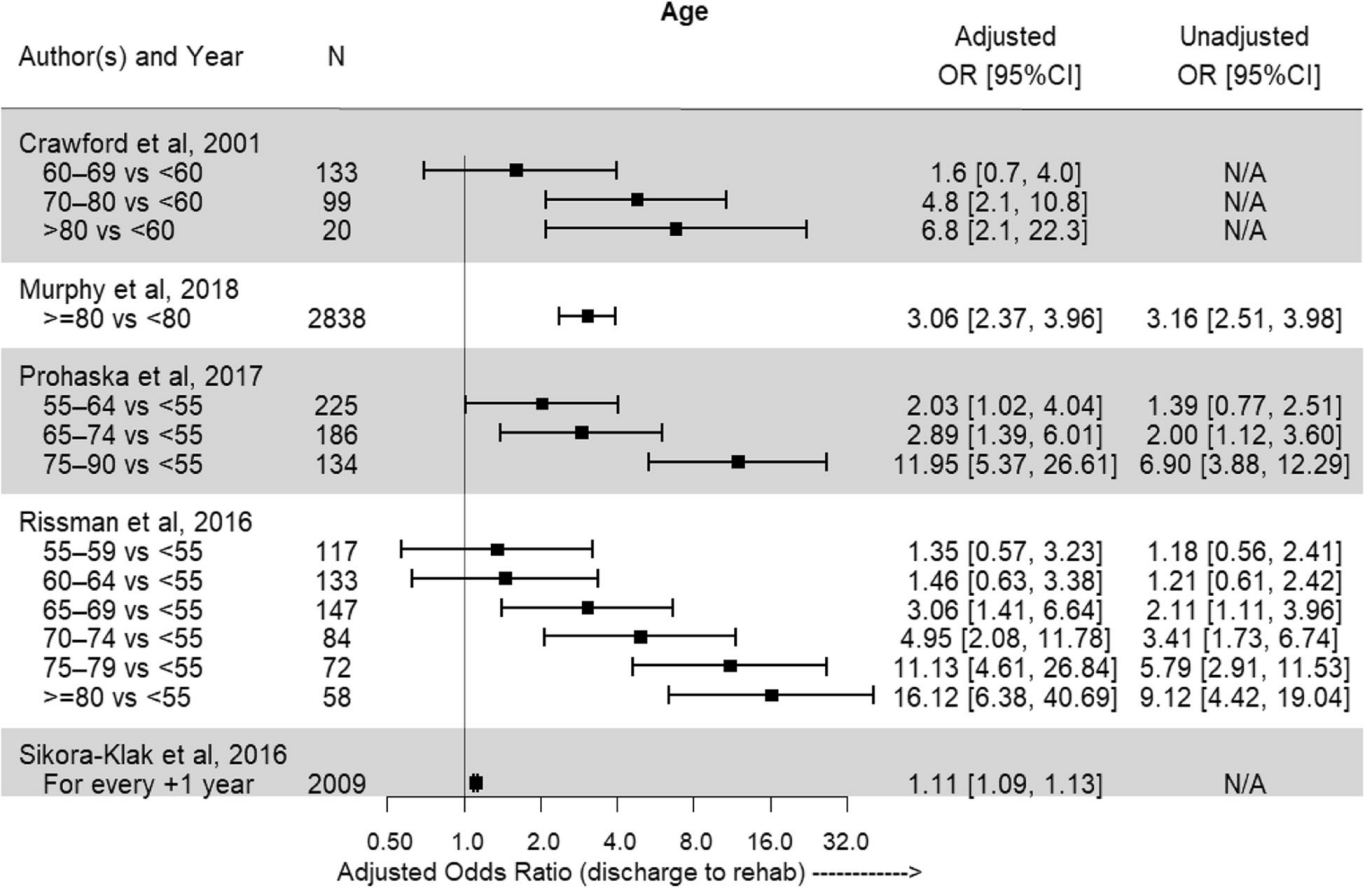
Adjusted odds ratios and 95%CI showing likelihood of discharge to IRF with older age. Unadjusted odds ratios with 95%CI are also reported

**Fig. 4:**
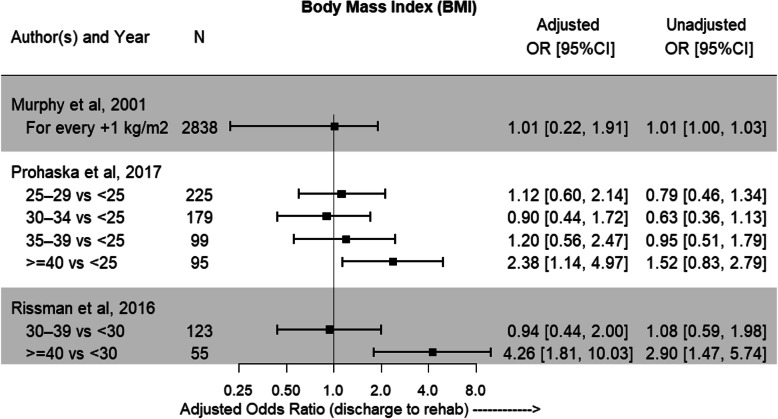
Adjusted odds ratios and 95%CI showing likelihood of discharge to IRF with increase in BMI. Unadjusted odds ratios with 95%CI are also reported

The patient factor gender was able to undergo meta-analysis in 4 studies [22–24, 27] to provide results of a combined effect on predictability of discharge destination. Being of female gender increased the likelihood of IRF discharge by 78% when compared to male gender (OR = 1.78; 95% CI = 1.43–2.20) (Fig. 2). The association between age and discharge destination was reported in 6 studies [20, 22–24, 26, 27]. Older age was predictive of IRF discharge in all included studies, with the greatest effect for those aged 75 years and older (Fig. 3). Increased BMI was also able to be included for review in 3 studies [22–24], with those in the severely obese (≥40 kg/m^2^) category having the highest likelihood of IRF discharge (Fig. 4).

#### Clinical factors

**Fig. 5:**
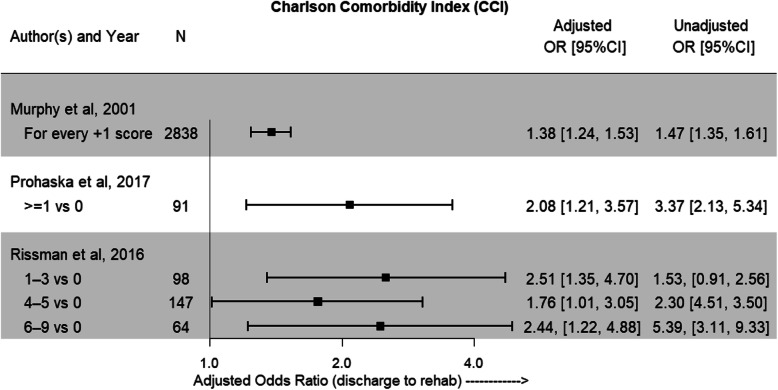
Adjusted odds ratios and 95%CI showing likelihood of discharge to IRF with higher CCI. Unadjusted odds ratios with 95%CI are also reported

Studies varied in their reporting of patient comorbidities. CCI was reported in 3 studies but due to methodological heterogenicity could not be meta-analysed [22–24]. The CCI quantifies an individual’s burden of disease and corresponding 1-year mortality risk, with a lower score equalling a lower risk [28]. Figure 5 shows a trend was towards a higher CCI being more predictive of IRF discharge.

#### Behavioural factors

**Fig. 6:**
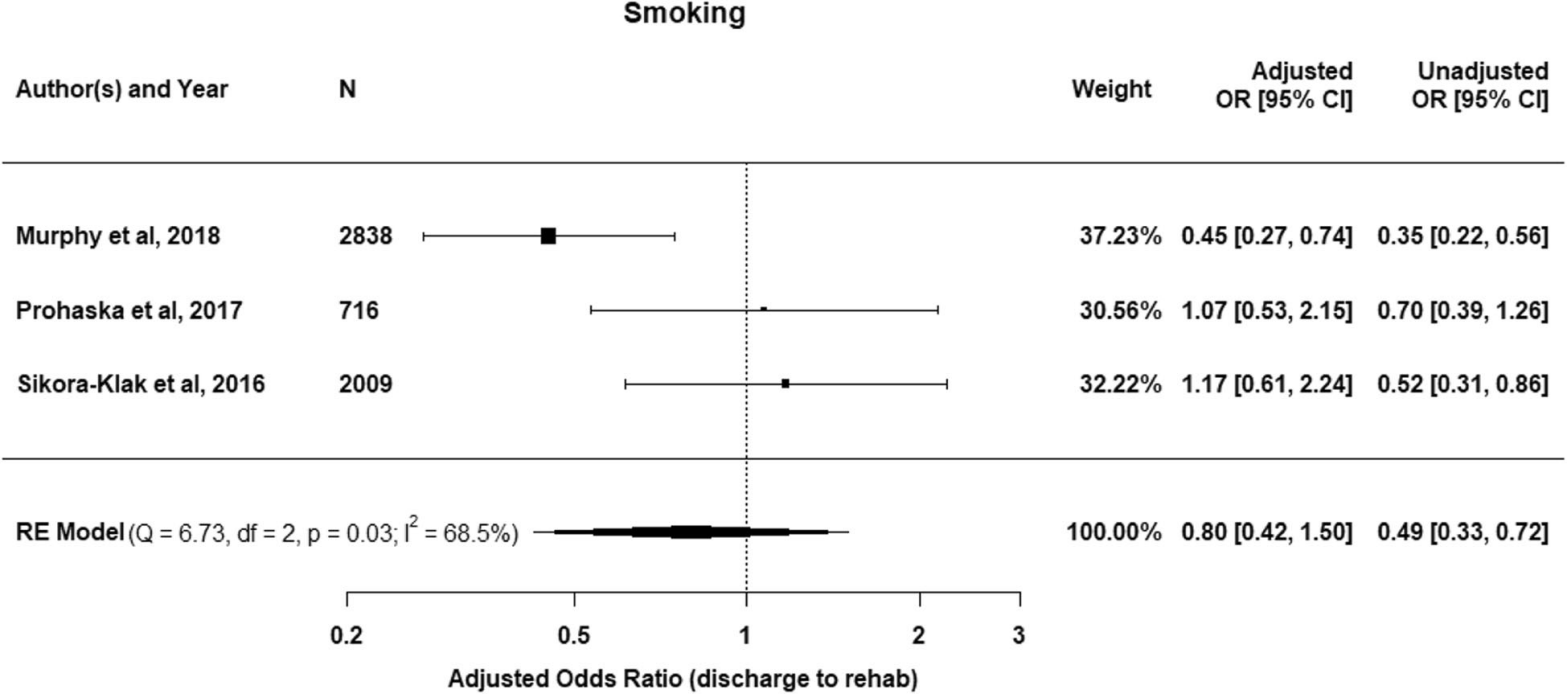
Meta-analysis showing the adjusted odds ratios and 95%CI of a random effects (RE) model for the effect of smoking on discharge to IRF. Unadjusted odds ratios with 95%CI are also reported

Smoking status (non-smoker or currently smoking) was reported on in 3 studies and was included in meta-analysis [22, 23, 27]. Smoking showed an overall decreased likelihood of IRF discharge (OR = 0.80; 95% CI 0.42–1.50), however, heterogeneity of the studies was moderate (*I*^*2*^ = 68.5%) (Fig. 6).

#### Patient reported outcome measures

**Fig. 7:**
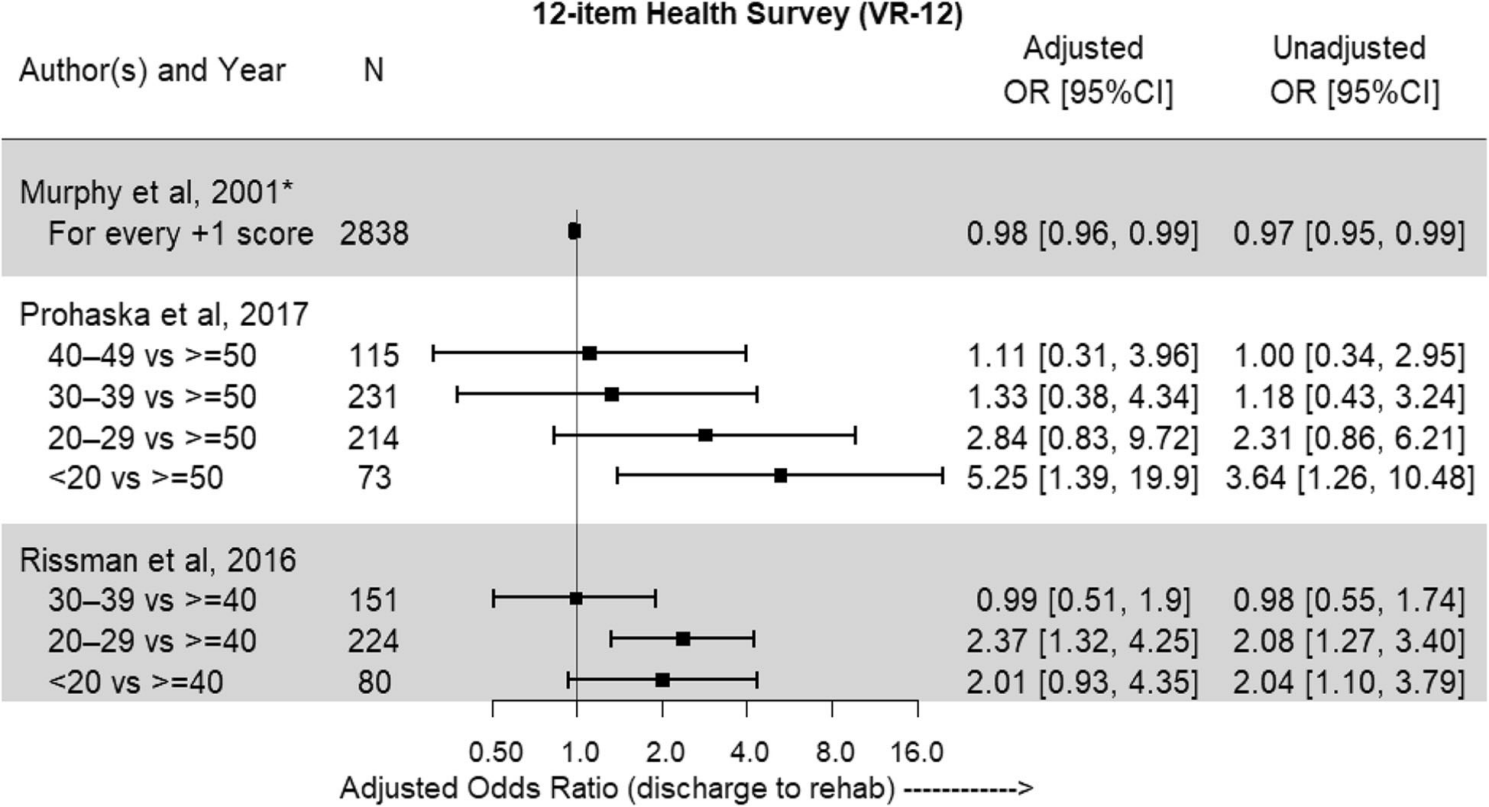
Adjusted odds ratios and 95%CI showing likelihood of discharge to IRF with lower VR-12 score. Unadjusted odds ratios with 95%CI are also reported. ^★^The SF-12 Health Survey was used.

A self-reported measure of physical function was assessed in 3 [22–24] of the 9 included studies, and due to the similarity of the design and scoring systems of the SF-12 and VR-12 it was decided to combine the results of these tools (Fig. 7) [29, 30]. Only 1 study, Prohaska et al., demonstrated a consistent association with IRF discharge related to lower self-reported physical function, with the other included studies showing the relationship to be more unclear.

## Discussion

This systematic review and meta-analysis demonstrates that it is difficult to develop predictive models for intrinsic patient factors associated with IRF discharge based on the current existing literature. The large degree of heterogeneity and wide variation of statistical analysis and reporting across the included studies precluded meta-analysis for all but 2 variables. Two included factors underwent meta-analysis, gender and smoking (Figs. 2 and 6). There was a clear association between female gender and likelihood of IRF discharge, however, the relationship between smoking status and IRF discharge was less certain due to the heterogeneity of the studies included in the smoking meta-analysis (*I*^*2*^ = 68.5%). For the other intrinsic patient factors included in this review, the strongest trends for discharge to IRF were older age, greater comorbidity or the severely obese. A worse self-reported physical function was not consistently associated with an increased risk for IRF discharge.

Although to our knowledge this is the first systematic literature review and meta-analysis where the primary outcome of interest is discharge destination, systematic reviews on patient factors predictive of increased length of hospital stay following TJA have been published [31, 32]. The patient risk factors found in this review that trended towards an increased likelihood of discharge to IRF, are similar to those reported for increased length of stay, including female gender, older age, increased comorbidities and higher BMI [31, 32].

A limitation of this review is that the studies included were mostly of retrospective design and all utilised large medical databases as the source of patient predictors. The use of large databases for analysis has been reported as having limitations such as coding bias [33]. Additionally, using a retrospective cohort design limits the investigation to only variables recorded at that time. It is likely that other surgical, psychological and sociological variables could be predictive of discharge destination, however, if this information is not available at the time of retrospective data collection then it will not be included in analysis. In terms of sociological factors, insurance status has been shown to be predictive of IRF discharge [34], however, due to the international variability of insurance models this was excluded from analysis.

One psychological variable that has been shown to be predictive of discharge destination following TJA is patient expectation [35]. Halawi et al. found that a patient’s pre-operative expectation of their discharge destination was the strongest predictor of actual discharge destination even when adjusted for other variables such as age and caregiver assistance. As this study did not separate TKA from total hip arthroplasty it was not eligible for inclusion in this review, but it does highlight the need for more studies incorporating patient belief systems into predictive modelling for IRF discharge.

With a growing body of evidence suggesting that IRF discharge following routine primary TKA is not superior to home discharge, further prospective high-quality studies investigating the patient factors that are predictive of discharge destination are needed. Previous studies have assessed patient factors by retrospectively accessing medical records, however, many of these are non-modifiable such as age and gender. With significantly increased costs associated with IRF when compared with home discharge after TKA, modifiable patient factors such as BMI and patient expectation should be given priority in future investigations.

## Conclusion

This systematic review and meta-analysis illustrates that although literature exists on investigating which intrinsic patient factors are predictive of IRF discharge, there is large variation in statistical methods and reporting. Female gender and smoking were two patient factors able to be included in this meta-analysis, with female gender shown to be predictive of IRF discharge, however, the relationship between smoking and discharge destination was less certain. There was also a trend for those of older age, increased comorbidity or in a severely obese category to have an increased likelihood of IRF discharge.

## Data Availability

All data generated or analysed during this study are included in this published article.

## Abbreviations

ASA: American Society of Anaesthesiologists
BMI: Body Mass Index
CCI: Charlson Comorbidity Index
CHARMS: Checklist for critical Appraisal and data extraction for systematic Reviews of prediction Modelling Studies
Hb: Hemoglobin
IRF: Inpatient Rehabilitation Facility
LOS: Length of Stay
PRISMA: Preferred Reporting Items for Systematic Reviews and Meta-Analyses
PROM: Patient Reported Outcome Measure
QUIPS: Quality In Prognosis Studies
ROM: Range of Motion
SES: Socio-economic status
SF-12: Short Form 12 item survey
TKA: Total Knee Arthroplasty
VTE: Venous Thromboembolism
VR-12: Veteran’s Rand 12 item survey
